# SNP Analysis Reveals Novel Insights into the Genetic Diversity of Colombian *Vaccinium meridionale*

**DOI:** 10.3390/genes16060675

**Published:** 2025-05-30

**Authors:** John Sepúlveda, Fernando Rondón González, Johana Carolina Soto Sedano, Ginna Patricia Velasco, Teresa Mosquera, María Cecilia Delgado, Gustavo Adolfo Ligarreto Moreno, Stanislav Magnitskiy, Yuranis Miranda, Luz Nayibe Garzón Gutiérrez

**Affiliations:** 1 Escuela de Biología, Facultad de Ciencias, Universidad Industrial de Santander, Bucaramanga 680002, Colombia; john.e.sepulveda@hotmail.com (J.S.); ferongon@uis.edu.co (F.R.G.); yuranismirandam@gmail.com (Y.M.); 2 Departamento de Biología, Facultad de Ciencias, Universidad Nacional de Colombia, Sede Bogotá, Bogotá 111321, Colombia; jcsotos@unal.edu.co; 3 Departamento de Agronomía, Facultad de Ciencias Agrarias, Universidad Nacional de Colombia, Sede Bogotá, Bogotá 111321, Colombia; velascoginna@gmail.com (G.P.V.); tmosquerav@unal.edu.co (T.M.); mcdelgadon@unal.edu.co (M.C.D.); galigarretom@unal.edu.co (G.A.L.M.); svmagnitskiy@unal.edu.co (S.M.)

**Keywords:** Andean blueberry, vaccinium, agraz, Ericaceae, population structure, single-nucleotide polymorphism, genotyping by sequencing

## Abstract

Agraz (*Vaccinium meridionale* Swartz) is a shrub native to the Neotropical region of South America, including Colombia, Ecuador, Venezuela, and Peru. Known for its edible fruits valued for their nutritional, nutraceutical, and medicinal properties, the species remains underexplored despite its potential. This research aimed to investigate the genetic diversity and population structure of agraz in Colombia, focusing on native individuals from Santander and commercial individuals from Boyacá and Cundinamarca, providing insights that can support conservation and genetic improvement efforts. **Methods**: In this study, genotyping by sequencing (GBS) was used to analyze genetic variation and population structure in *V. meridionale*. The sequencing data were aligned to the *V. corymbosum* cv. Draper v1.0 reference genome. The obtained single-nucleotide polymorphisms (SNPs) were employed to evaluate genetic diversity, population differentiation, and inbreeding coefficients, with measures such as expected heterozygosity and F-statistics providing insights into population structure and genetic composition across regions. **Results**: A total of 12,910 SNPs were obtained, and the results revealed moderate genetic diversity within the agraz populations, characterized by an expected heterozygosity (He) of 0.3586. A negative Fis value indicated an excess of heterozygosity and low genetic differentiation among the sampled regions. Population structure analysis identified three distinct subpopulations, with Subpopulation 3 exhibiting the most unique genetic composition. **Conclusions**: This study provides the first genetic diversity analysis of *V. meridionale* in Colombia using the GBS approach. The findings contribute to the understanding of the species’ genetic variability and offer valuable information for conservation strategies, genetic improvement and breeding programs to enhance its agricultural potential and ensure the sustainable utilization of agraz resources.

## 1. Introduction

Agraz (*Vaccinium meridionale* Swartz) is an evergreen and wild tetraploid (2n = 4x = 48) species belonging to the Ericaceae family. The fruit produced by the *V. meridionale* plant is commonly known in various regions as mortiño, vichachá, Colombian blueberry or Andean blueberry [1,2,3]. Agraz typically grows as a shrub, but tree-like habits have also been identified [4]. Research in the last decade has highlighted the nutritional and nutraceutical properties of *V. meridionale* fruit, which has given it the nature of a promising plant and a potential role promoting health by preventing diseases such as cancer and Alzheimer’s disease, as well as benefits regulating blood glucose and slowing down aging [5,6,7,8]. In the Andean high forest, *V. meridionale* plays an ecological role maintaining pollinator populations, providing food for wild fauna, and preventing soil erosion [9,10]. Additionally, it is of interest to plant breeders due to the high number of flowers on each branch and its primarily monopodial morphology, which is useful for mechanical harvesting [11]. It is native to the neotropical mountainous regions between 2000 and 3000 m above sea level (m a.s.l.) in countries such as Colombia, Ecuador, Venezuela, and Peru [12]. In Colombia, *V. meridionale* is mainly distributed in the departments of Antioquia, Boyacá, and Cundinamarca, with records of both native and cultivated populations [4,13]. However, in the department of Santander, despite the favorable conditions of the high Andean Forest, information about the composition and distribution of natural populations of this species is limited, representing a challenge to understand its genetic diversity and potential sustainable use. *V. meridionale,* as part of the high Andean Forest ecosystems, faces significant threats due to anthropogenic intervention, including the expansion of the agricultural frontier, as well as the establishment of human settlements in rural areas [4,14]. Other factors such as mining and climate change can also threaten the stability of these ecosystems [15]. Traditionally, *V. meridionale* has been exploited locally through the direct harvest of fruits in wild populations. Some farmers have even transplanted plants from other regions or departments to establish crops. This practice could also be adversely affecting the genetic basis of the species, leading to a possible loss or decrease in genetic diversity [16,17]. Likewise, no studies have been conducted in the department of Santander to determine the distribution of *V. meridionale*, nor to evaluate the genetic diversity and the state of genetic differentiation of the native populations [18,19]. Additionally, knowledge of genetic diversity is essential to understand and address the challenges of conservation and domestication of a wild species like *V. meridionale*. Similarly, identifying certain genome regions associated with traits of interest can be useful for carrying out breeding programs through crosses or obtaining fertile hybrids, as has been performed with *V. meridionale* and *V. corymbosum* [18], *V. vitis-idaea* [11], and *V. macrocarpon* [19]. These programs aim to improve pollen production and quality, plant vigor, and fertility.

In crops of the genus *Vaccinium*, such as *V. macrocarpon,* genotyping approaches such as genotyping by sequencing (GBS) have been employed [20,21]. GBS is a genomic analysis technology that enables the identification of genetic variations across the entire genome by generating a matrix of thousands of single-nucleotide polymorphisms (SNPs) [22]. GBS facilitates genetic fingerprinting analysis and the exploration of species diversity and population structure at low cost [23,24,25]. In this study, GBS was conducted to evaluate the genetic diversity and population structure of *V. meridionale* in Colombia for the first time. The analysis included a population of 72 native individuals from Santander, and 51 commercial individuals of native populations from Boyacá and Cundinamarca, for a total population of 123 genotypes. We expected the genetic diversity and population structure of *V. meridionale* in Colombia to differ between native and commercial populations, with native populations from Santander exhibiting higher genetic diversity compared to commercial populations from Boyacá and Cundinamarca. These differences would provide valuable insights into the species’ genetics and population dynamics.

## 2. Materials and Methods

### 2.1. V. meridionale Plant Material Collection

The collection of *V. meridionale* materials were carried out in accordance with Amendment 31 to the framework contract for Access to Genetic Resources and their derivatives No. 121 dated 22 January 2016, signed between the Colombian Ministry of Environment and Sustainable Development and the Universidad Nacional de Colombia, as well as the contract for Access to Genetic Resources and their derivatives No. 338-File RGE403 dated 15 June 2022, signed between the Colombian Ministry of Environment and Sustainable Development and the Universidad Industrial de Santander.

**Figure 1:**
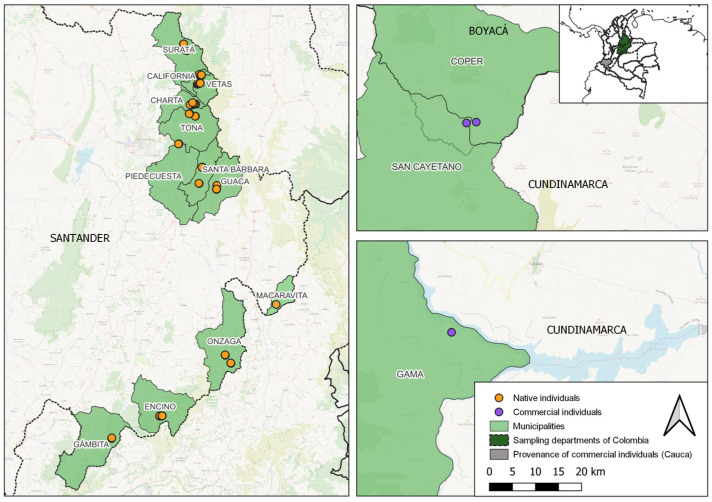
Geographic location of the natural and commercial *Vaccinium meridionale* individuals analyzed. Geographic locations where the 123 *V. meridionale* materials included in the study population were collected.

Exploratory field trips were conducted to collect plant material from native populations of *V. meridionale* in 12 municipalities located in the department of Santander, Colombia which were, California, Charta, Encino, Gambita, Guaca, Macaravita, Onzaga, Piedecuesta, Santa Bárbara, Suratá, Tona, and Vetas (Figure 1 and Supplementary Table S1). Plant identification was confirmed using the varietal descriptors of Medina et al. [26,27]. In each municipality, depending on availability, up to 10 individuals were selected and collected, and biogeographical data were recorded for each site. Young leaves free of mechanical damage, pathogens, or insect injury were collected and immediately placed in liquid nitrogen. Samples were then stored at −80 °C. In total, 123 DNA samples from 123 individuals of *V. meridionale* were obtained at altitudinal ranges varying from 2269 to 3031 m a.s.l. in diverse environments, including sites exposed to direct sunlight, vegetation with multiple plant species providing partial shade, and oak forests (*Quercus* sp.) with leaf litter on the ground almost entirely under shade.

For the purposes of this study, plants classified as “native” were those sampled directly from wild populations in natural forest environments, without any record of human-mediated propagation or relocation. In contrast, individuals classified as “commercial” refer to native plants that local farmers have extracted from their natural habitats and cultivated in managed plots. These plants have not undergone formal domestication or breeding programs but are maintained outside of their original ecological context. Samples of these commercial individuals were collected from the municipalities of Coper (Boyacá), Gama and San Cayetano (Cundinamarca).

### 2.2. V. meridionale DNA Extraction

DNA extraction was performed following a combination of the protocols by Azmat et al. [28] and Inglis et al. [29] with some modifications. Two grams of finely macerated leaf tissue with liquid nitrogen was taken. The pre-wash protocol with sorbitol was followed according to Inglis et al. [29], with centrifugation set at 5000× *g* for 5 min at 18 °C. Subsequently, the plant DNA extraction protocol with CTAB by Azmat et al. [28] was followed and the final pellet resuspension was performed with 60 µL of TE buffer. The quality of the DNA was verified using a Nanodrop 2000 spectrophotometer (Thermo Fisher Scientific, Waltham, MA, USA) and quantified with NanoDrop and Qubit 4 (Thermo Fisher Scientific, Waltham, MA, USA). The integrity of each DNA sample was verified on 1% agarose gels stained with SYBR Safe and visualized under UV light. Finally, the samples were stored at −20 °C.

### 2.3. Library Preparation and Genotyping by Sequencing (GBS)

Libraries were prepared and GBS data were generated by the Elshire Group in New Zealand, following the method of Elshire et al. [22]. A total of 100 ng of gDNA was used with 3.6 ng of adapters. The gDNA was restricted with *ApeKI* enzyme, and the library was amplified with 18 PCR cycles. All samples were sequenced in two lanes of the Illumina Hi-seq platform (Illumina, Inc., San Diego, CA, USA). The GBS analysis pipeline GBSV2 [30,31], was applied for the SNP calling. Currently, *V. meridionale* does not have a reference genome, and so the genome of the tetraploid species *V. corymbosum* cv Draper, available at the National Center of Biotechnology under the bioproject PRJNA494180 [32], was used for mapping reading. Although *V. corymbosum* and *V. meridionale* belong to different taxonomic sections (Cyanococcus and Pyxothamnus, respectively), phylogenetic studies have shown that species across sections can be closely related [33,34]. Furthermore, fertile intersectional hybrids between these two species have been reported [18], indicating genetic compatibility. The *V. corymbosum* cv. Draper genome has also been widely used as a reference for GBS studies in various *Vaccinium* species due to its quality and genomic resources [35,36]. Finally, SNP filtering was performed using the GBS pipeline implemented in the TASSEL 5.2.16 software [30]. A minor allele frequency (MAF) of 1% (0.01) was adjusted, SNPs with more than 30% missing data in the proposed genotypes were removed, and genotypes with 70% missing data were identified and eliminated. Additionally, filters were applied to remove INDELS, multiallelic SNPs, and monomorphic SNPs. The original matrix, comprising 123 samples, exhibited an overall missing data rate of 27.10%. Regarding sequencing quality control thresholds, minimum base quality scores of Q20 and a minimum read depth of 10 reads were applied during SNP calling.

### 2.4. SNP Characterization

SNP markers were analyzed to determine their transition/transversion rate. The SNPs were classified based on their genomic location, including exons, introns, intergenic regions, the 3′ UTR, and the 5′ UTR. This classification was performed using the GenomicRanges [37], rtracklayer [38], and VariantAnnotation [39] packages in R (version 4.4.1). The SNP density plot was generated using the CMPlot R package (version 4.4.1) [40].

### 2.5. Population Structure Analysis and Genetic Diversity Parameters

The population structure of the 123 genotypes of *V. meridionale* was evaluated using a genetic structure analysis of admixture using sNMF v.1.2 with the Landscape and Ecological Association (LEA) R package [41]. Different numbers of subpopulations (K) were analyzed, from 2 to 8 (with 100 replicates and 10,000 α each), and the K value was selected using the computational criterion of cross-entropy 2–8 established by the package. The assignment of each genotype to a subpopulation was made with a membership probability of at least 75%. The genetic diversity of *V. meridionale* was analyzed using the Hierfstat Package v 0.5.11 [42]. The assignment of each genotype to a subpopulation was made with a membership probability of at least 75%. The gene diversity (D_ST_, Diversity among Subpopulations) and Corrected Gene Diversity (D_STP_, Diversity of Subpopulations Total Proportion) were calculated among individuals, overall gene diversity (H_T_) and corrected gene diversity (H_TP_) among populations, the fixation index (F_ST_) and its corrected version (F_STP_), and the inbreeding coefficient (F_IS_). Overall observed heterozygosity (Ho) and genetic diversity (Hs) within populations were estimated based on mean allele frequency. Furthermore, a dendrogram was constructed using the Neighbor-Joining (NJ) algorithm in the ggtree v 3.6.2 package [43] based on Nei’s genetic distances. Finally, a kinship analysis was performed based on the Van Raden distances between the genotypes [44]. This analysis utilized the GAPIT function from the R package GAPIT3 v 3.5.0 [45]. The resulting kinship matrix was then visualized through a heatmap generated using the pheatmap package v 1.0.12 [46].

## 3. Results

### 3.1. SNP Genotyping and Characterization

**Figure 2:**
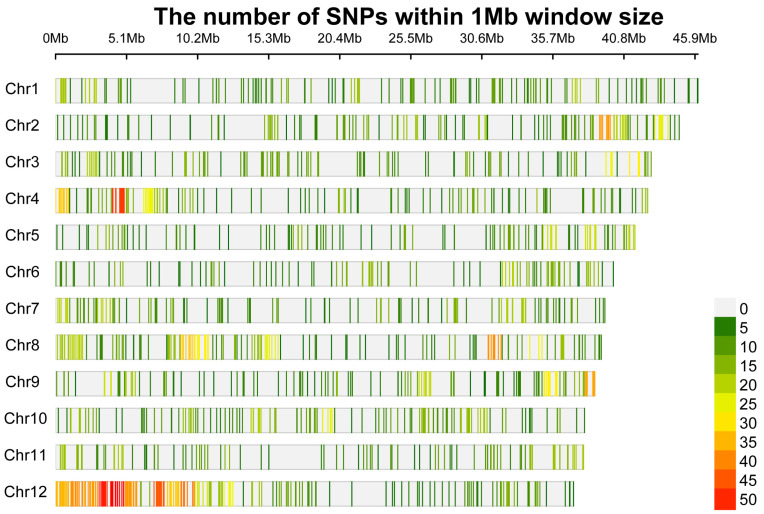
GBS-SNP density plot. Physical map of the number of SNPs within *Vaccininum meridionale* of a 1000 kb window size on 12 chromosomes. The color gradient represents the number of SNPs within each window, with darker colors indicating higher SNP density. A color scale indicating the corresponding SNP counts is shown in the bottom right corner.

A total of 12,910 SNPs with genome positions from the reference genome of *V. corymbosum* cv. Draper v1.0 were identified from 123 genotypes of *V. meridionale*. Of these, 4018 SNPs were mapped onto the 12 chromosomes with an average density of 1 SNP every 0.01 kb, distributed across all chromosomes. The chromosome with the highest SNP density was Chromosome 12, with 509 SNPs per 62,929 bp, whereas Chromosome 4 had the lowest SNP density, with 11 SNPs per 221,683 bp (Figure 2); the remaining 8892 SNPs were distributed across 302 scaffolds of the reference genome. From the total SNP dataset, 63.1% (8149) were transitions, while 37.2% (4761) were transversions, resulting in a transition-to-transversion ratio of 1.7. Regarding genomic location, 45.3% (5855) of the SNPs were found in exons, 26.9% (3472) in introns, 22.6% (2913) in intergenic regions, 3.6% (463) in the 3′ UTR, and 1.6% (207) in the 5′ UTR (Supplementary Figure S1).

### 3.2. Population Structure Analysis

**Figure 3:**
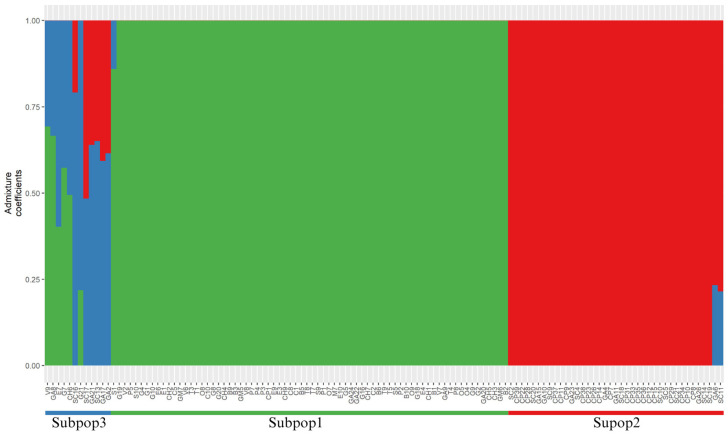
Genetic population structure. Genetic structure and differentiation of 123 *V. meridionale* accessions estimated by LEA admixture coefficient analysis with three subpopulations (K).

The K values tested from 2 to 8 using cross-entropy analysis in the LEA R package v 3.14.0 indicated that K = 3 was the optimal value to explain the population structure (Figure 3). Most genotypes were clustered into Subpopulation 1 (Subpop1), represented by a green color, which included 72 genotypes. Subpopulation 2 (Subpop2) included 39 genotypes and was indicated by red color, while Subpopulation 3 (Subpop3) contained the remaining 12 genotypes. In Subpop1, nearly all individuals from the department of Santander were grouped together with a few genotypes from the municipality of Gama (Cundinamarca) and Coper (Boyacá). In contrast, Subpop2 predominantly consisted of commercial individuals from Boyacá and Cundinamarca, whereas Subpop3 contained a mix of samples from all three departments: Santander, Boyacá, and Cundinamarca.

### 3.3. Genetic Diversity Analysis

**Figure 4:**
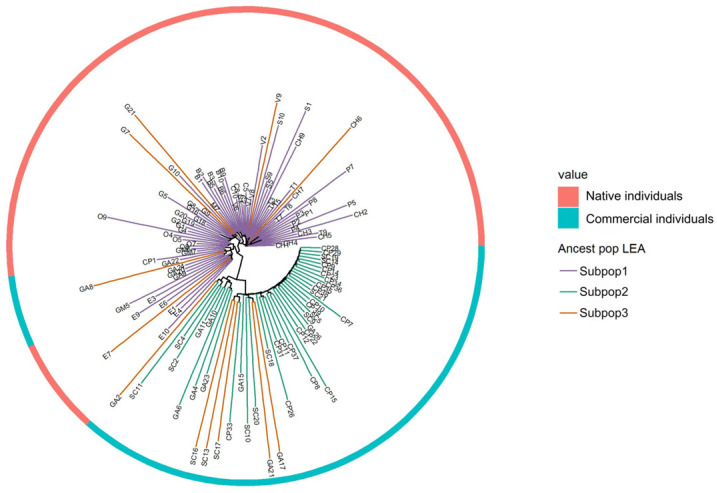
Neighbor-Joining dendrogram illustrating the genetic relationships among 123 *Vaccinium meridionale* genotypes. Within the circle, the NJ dendrogram displays Nei’s distances of the population. The colors purple, green and orange indicate the correspondence of each *V. meridionale* genotype to the subpopulations identified by Subpop1, 2, and 3, respectively.

The previously established set of subpopulations were used to determine the different groups of genotypes. The phylogenetic relationship among genotypes was explored by constructing a Neighbor-Joining dendrogram with SNP data. Distinct genetic groups were identified, predominantly reflecting the subpopulations determined by the population structure analysis using the LEA package v 3.14.0 (Figure 4). Moreover, the inner ring of the graph highlights a pronounced differentiation between the wild and wild commercial groups, indicating that the sample origin is an important factor influencing genetic structure.

Genetic diversity parameters were evaluated in the entire population as well as in the three subpopulations identified by the genetic structure analysis. The overall observed heterozygosity (Ho) for the entire population was 0.4653, notably higher than the expected heterozygosity (He) of 0.3586, resulting in an inbreeding coefficient (F_IS_) of −0.2975 (Table 1). This negative Fis value indicates an excess of heterozygotes in the population, suggesting low levels of inbreeding and possibly outcrossing. The total gene diversity (H_T_) was 0.3682, with a genetic differentiation (F_ST_) of 0.0259, indicating that only 2.59% of the genetic variation is due to differences among populations. The corrected gene diversity (H_TP_) was 0.3729, and the corrected genetic differentiation (F_STP_) was 0.0383, suggesting that the genetic variation remains low among subpopulations even when accounting for potential biases. The overall gene flow (D_EST_) of 0.0223 indicates a moderate level of gene exchange among subpopulations, further supporting the idea of genetic similarity among them.

Subpop1 had the highest observed heterozygosity (Ho = 0.5678) compared to its expected heterozygosity (He = 0.3654), yielding a corresponding F_IS_ of −0.554. Subpop2 showed slightly lower observed heterozygosity (Ho = 0.5592) and expected heterozygosity (He = 0.3586), resulting in a F_IS_ of −0.559. These negative F_IS_ values indicate an excess of heterozygotes and reduced inbreeding within these subpopulations. Conversely, Subpop3 displayed an observed heterozygosity of 0.2689 and an expected heterozygosity of 0.3602, yielding a positive F_IS_ of 0.253. This suggests a moderate level of inbreeding within Subpop3, with fewer heterozygotes than expected. On the other hand, Nei and F-Statistics for subpopulations of *V. meridionale* genotypes (Table 2) indicate moderate genetic differentiation between the subpopulations, with F_ST_ values ranging from 0.050 to 0.060, suggesting that around 5–6% of the genetic variation is due to differences among these subpopulations. The genetic distance (Nei’s) values range from 0.029 to 0.036, indicating a moderate level of genetic diversity among them. Subpop1 and Subpop3 are the most genetically similar, while Subpop1 and Subpop2 show the highest differentiation. Overall, these results imply that while there is some genetic differentiation among the subpopulations, they still share a considerable amount of genetic similarity, reflecting moderate gene flow and common ancestry within the studied populations.

## 4. Discussion

Understanding the genetic diversity within wild plant species is essential for their survival, their adaptation to environmental changes, and the advancement of crop improvement and breeding efforts [47,48]. *V. meridionale*, a wild berry native to the Andean region of South America, has gained attention for its potential as a cultivated crop and its notable health benefits. The plant exhibits significant phenotypic plasticity in natural populations and displays variations in morphological traits [49]. Its fruits are rich in antioxidants, particularly anthocyanins and polyphenols, enhancing its nutritional value [6,50,51]. Traditionally, *V. meridionale* has been used medicinally for digestive, genitourinary, and metabolic disorders [5,51]. Furthermore, recent studies highlight its potential for cultivation and breeding programs due to its unique traits and ability to hybridize with highbush blueberries [18,19]. In this study, we present the first analysis of genetic diversity and population structure of *V. meridionale* using SNP molecular markers. We used 72 native individuals or genotypes from the department of Santander, along with 51 commercial individuals or genotypes from Boyacá and Cundinamarca, located in the Central-Eastern region of Colombia. By employing GBS, we identified a total of 12,910 high-quality SNPs, yielding an average density of 1 SNP per 0.01 kb. These results are comparable to those reported for other species within the *Vaccinium* genus, which also exhibit high SNP densities, despite variations in sample size and the total number of SNPs when using the same GBS technique. For instance, Alam et al. [52] identified 1586 high-quality SNPs in lingonberry (*V. vitis-idaea*). Campa and Ferreira et al. [21] reported 5255 SNPs from an analysis of 70 individuals, including *V. corymbosum*, *V. virgatum*, *V. macrocarpon*, and *V. uliginosum*. In contrast, Kulkarni et al. [35] identified 92,048 SNPs in 99 individuals among *V. corymbosum*, *V. darrowii*, *V. virgatum*, and *V. tenellum*, while Manzanero et al. [36] found 60,518 SNPs in an analysis of 195 individuals of *V. corymbosum*, *V. boreale*, *V. darrowii*, *V. myrsinites*, and *V. tenellum*. On the other hand, the high proportion of SNPs identified in coding regions through GBS (45.3%) highlights its potential as a powerful tool for Genome-Wide Association Studies (GWAS). This is particularly relevant for identifying allelic variants associated with commercial traits in *V. meridionale*, as SNPs within coding sequences are more likely to have functional implications. Similarly, in *V. corymbosum,* GWAS has been used to identify polymorphisms associated with agronomic traits. For instance, previous studies have detected genomic regions related to fruit quality [53] and volatile organic compounds [54], along with specific SNPs on Chromosome 4 associated with phenological traits such as maturation date [55]. This demonstrates the relevance of SNPs in coding regions for understanding the genetic basis of key traits in *Vaccinium* species.

It is important to acknowledge that *V. meridionale* is a tetraploid species, which presents inherent challenges for SNP analysis using GBS [53]. In polyploid species, traditional SNP calling pipelines optimized for diploid genomes often fail to capture allele dosage accurately and may confound homologous variation with true polymorphisms [53]. These limitations can result in biased allele frequency estimates and hinder the detection of meaningful genetic associations. Studies on autotetraploid *Vaccinium* species, such as *V. corymbosum* [53,54], which also used the *V. corymbosum* reference genome, have demonstrated that explicitly modeling allelic dosage significantly improves the power and resolution of GWAS analyses. However, such approaches require high sequencing depth, bioinformatic tools capable of modeling polyploid inheritance, and complex statistical models—conditions that are not yet fully optimized or widely accessible for non-model species like *V. meridionale*. Therefore, we adopted a diploidized analysis strategy which, while not modeling dosage explicitly, remains a widely accepted approximation in early genomic studies of polyploid species [53]. This approach is particularly appropriate when the main goal is to explore population structure and diversity patterns in the absence of more specialized polyploid-oriented resources. As sequencing strategies and polyploid-specific tools continue to evolve, future studies will be better positioned to refine SNP interpretation and adopt more realistic models tailored to the genetic architecture of *V. meridionale*.

The complete population has a moderate genetic diversity (Hs = 0.359) and an excess of heterozygosity (Ho > Hs). This pattern may be a result of the introgression of alleles from another population through gene flow explained by the F_ST_ value (Table 1) and the genotypes with mixed blue with red and green colors in Figure 3, or natural selection. Moderate-to-high levels of diversity are relatively common in *Vaccinium* species. High heterozygosity was observed across populations of *V. macrocarpon* (Hs = 0.99) and *V. oxycoccos* (Hs = 0.71) using 32 simple sequence repeat (SSR) markers [56]. Similarly, tetraploid species like *V. angustifolium* (Hs = 0.62) and *V. corymbosum* (Hs = 0.76) exhibit high heterozygosity according to isozyme analysis [57], and even closely related species in South America, such as *V. floribundum*, demonstrate significant value of genetic diversity (Hs = 0.73) using SSR markers [58]. This high diversity in *Vaccinium* species could be related to their wide geographic distribution, multiple pollination mechanisms, high levels of hybridization, polyploidy, or gene flow. These characteristics are present in *V. meridionale* [1,4,11]. However, some subpopulations deviate from this general pattern, suggesting local demographic or ecological effects. The positive inbreeding coefficient (FIS) detected in Subpop3 contrasts not only with the other subpopulations, but also with the overall pattern observed in the complete dataset, where a negative FIS indicates an excess of heterozygosity (Table 1). This divergence in Subpop3 can be interpreted considering the reproductive biology of *V. meridionale*, which exhibits a facultative xenogamy system that allows for both cross- and self-pollination depending on environmental conditions [1]. In small, isolated populations or habitats with limited pollinator availability, such as regeneration areas, self-pollination becomes a crucial reproductive assurance strategy, leading to increased homozygosity and consequently higher FIS values [1].

The population structure analysis revealed three genetic subpopulations of *V. meridionale* within native and commercial genotypes (Figure 3). The high genetic differentiation value detected between Subpop1 and Subpop2 (F_ST_ = 0.06) suggests that geographic distances might explain the patterns of genetic structure found in this study. Almost all the genotypes in Subpop1 represent native individuals collected in Santander, with a few individuals from commercial genotypes, while Subpop2 is represented only by commercial individuals. It is important to note that these commercial materials lack an accurate record of their origin, as they are materials that farmers harvest and extract from their natural sites to plant near their homes or on their farms, although most were likely sourced from the Cauca department, specifically the municipality of Caloto in the southwest of Colombia. Geographic distance and natural barriers such as the Magdalena River, the Andean forest, human settlements, and the Central and Eastern Andes may have contributed to the genetic differentiation observed between these subpopulations [59,60,61,62]. Although Subpop3 was identified as a distinct cluster in the population structure analysis, the NJ dendrogram (Figure 4) and kinship analysis based on VanRaden distances (Supplementary Figure S2) reveal that individuals from this group display an intermediate genetic composition between Subpop1 and Subpop2. Therefore, Subpop3 represents a genetic admixture rather than a completely distinct biological group, combining genetic backgrounds from both native and commercial sources.

The fact that farmers have introduced individuals from another department and cultivated them in Boyacá and Cundinamarca could lead to an effect of “unconscious selection”, whereby humans inadvertently impact the genetic pool of a species in a population [63]. This could be due to selecting plants with beneficial traits for cultivation or domestication, such as easier harvesting or better taste [63,64]. The mixed color (blue and red) observed in Subpop3 could be a result of the initial mixing process between plants from Cauca and those in the other departments such as Santander. Future studies are encouraged to incorporate ethnobotanical surveys and documentation of farmers’ selection practices to corroborate the potential role of unconscious selection in shaping the genetic diversity and structure of cultivated *V. meridionale* populations.

In this study, we used SNPs to analyze the population structure and genetic diversity of 123 *V. meridionale* genotype samples. In similar studies, the implementation of agro-morphological or phenotypic traits is commonly employed to enhance the analysis [21,52,65]. However, *V. meridionale*, despite its various uses in local cultures as a wild plant, has not undergone a domestication process. Consequently, it does not have a certified seed recognized by the Instituto Colombiano Agropecuario (ICA) or a germplasm bank. Additionally, the geographical distribution of this plant remains largely unknown, particularly in the department of Santander. Before this study was conducted, the Biodiversity Information System of Colombia (SiB Colombia database) [13] recorded 96 occurrences of *V. meridionale* in the department of Santander, spanning an altitudinal range of 2300 to 3959 m a.s.l, with data from 1927 to 2020. The total number of municipalities with records of this species in Santander were: Vetas (2), California (3), Concepción (24), Encino (3), Carcasí (9), Onzaga (46), and Girón (1). Records in the herbariums of the Universidad Industrial de Santander (UIS herbarium) and the Universidad Nacional de Colombia (COL herbarium), as well as the work by Medina et al. [26], documented records in California, Onzaga, and Vetas. This study confirms the presence of the species in other areas within Santander, such as Suratá, Santa Bárbara, Charta, Tona, Guaca, Piedecuesta, Gambita, and Macaravita, expanding the dispersal range of the species and demonstrating that it is possible to find it in different regions of the high Andean Forest. In these municipalities, the species was mainly found in mountainous regions, rural areas far from urban centers, pasture environments, under oak forests, or in patches of secondary forest, with varying light conditions ranging from full sunlight to partial shade. According to Luteyn [66], plants of the Ericaceae family are not highly tolerant of extreme heat or freezing temperatures, which aligns with the altitudinal range and climatic conditions observed for these municipalities according to the Instituto de Hidrología, Meteorología y Estudios Ambientales (IDEAM, Institute of Hydrology, Meteorology, and Environmental Studies). Despite these records, it was noted that in some municipalities, it was not common to find plants less than 5 m apart from each other, and even in large, forested areas it was not the predominant species. Therefore, it could be inferred that the populations might be dispersed and potentially isolated, suggesting that the species could face challenges in pollination, reproduction, and dispersal. This could be due to habitat fragmentation or specific environmental factors in the region that limit the species’ expansion. Furthermore, Luteyn [66] also mentions that although species of this family can grow under shaded environments, they are less likely to flower or fruit under these conditions. Complementarily, studies by Lobos et al. [67] and Dyukaryeva and Mallik [68] on plants within the genus *Vaccinium* indicate that shading not only delays flowering and reduces the number of flowers per plant, but also affects the fruit’s nutritional and antioxidant properties.

Our results align with the findings of previous authors, as most of the plants bearing fruit or flowers were found in environments with partial shade or direct sunlight in open or pasture areas. In contrast, most of the plants recorded under shade in oak forests lacked reproductive structures, preventing fruit collection. This species was not found to be a cultivable commercial crop in any of the evaluated municipalities, despite its documented uses [4,5,69]. Some of the consulted guides and community members were unaware of its potential as a commercial resource, often eradicating it due to its morphological similarity to *Pernettya prostrata* (Ericaceae), a toxic plant known as reventadera, borrachero, or mortiño venenoso [70,71].

## 5. Conclusions

Our results indicate that the populations and individuals of *V. meridionale* collected in Colombia exhibit moderate genetic diversity, with three genetic subpopulations. This provides a valuable framework for the development of conservation strategies aimed at preserving the genetic diversity of *V. meridionale.* Furthermore, they offer a practical resource for breeding programs by facilitating the strategic selection of genetically diverse parental lines to maximize heterosis and trait improvement. The high proportion of SNPs located within coding regions further enhances the utility of this dataset for future GWAS, which may identify allelic variants associated with commercially important traits such as fruit quality, antioxidant content, and environmental adaptability.

By providing foundational genomic resources and detailed insights into the population structure of *V. meridionale*, this plant has potential as a crop and is an opportunity to enhance biodiversity, support local economies, and encourage sustainable agricultural practices in the Andean region by fostering awareness and promoting the cultivation of this valuable berry. The future of *V. meridionale* relies on recognizing its value and implementing strategies that ensure its preservation and sustainable utilization for generations to come.

## Figures and Tables

**Table 1:** Genetic diversity parameters for *Vaccinium meridionale* genotypes collected in Colombia.

Subpopulation	Number of Individuals	Ho	He	F_IS_	H_T_
Complete	123	0.4653	0.3586	−0.2975	0.3682
Subpop1	72	0.5678	0.3654	−0.554	0.3654
Subpop2	39	0.5592	0.3586	−0.559	0.3586
Subpop3	12	0.2689	0.3602	0.253	0.3603

**Table 2:** Nei and F-Statistics for subpopulations of *Vaccinium meridionale* genotypes collected in Colombia.

Subpopulation Comparisons	F_ST_	Nei
Subpop1 vs. Subpop2	0.060	0.036
Subpop1 vs. Subpop3	0.050	0.029
Subpop2 vs. Subpop3	0.053	0.030

## Data Availability

The original contributions presented in this study are included in the article/Supplementary Materials. Further inquiries can be directed to the corresponding author.

## Supplementary Materials

The following supporting information can be downloaded at: https://www.mdpi.com/article/10.3390/genes16060675/s1. Figure S1: SNP classification. Figure S2: Kinship matrix among the 123 *V. meridionale* cultivars based on Van Raden distances. Table S1: Biogeographic data of sample collection.

## References

[1] Chamorro F.J., Nates-Parra G. (2015). Biología floral y reproductiva de *Vaccinium meridionale* (Ericaceae) en los Andes orientales de Colombia. Rev. Biol. Trop..

[2] de Valencia M.L.C., de Lozano N.B. (1995). Anatomia del fruto del “Agraz” *Vaccinium meridionale* Swartz. Acta Biol. Colomb..

[3] Redpath L.E., Aryal R., Lynch N., Spencer J.A., Hulse-Kemp A.M., Ballington J.R., Green J., Bassil N., Hummer K., Ranney T. (2022). Nuclear DNA Contents and Ploidy Levels of North American Vaccinium Species and Interspecific Hybrids. Sci. Hortic..

[4] Magnitskiy S. (2023). Native Plants from the Genus Vaccinium in Colombia and Their Potential Uses. A Review. Rev. Colomb. Cienc. Hortícolas.

[5] Abreu O.A., Barreto G., Prieto S. (2014). Vaccinium (Ericaceae): Ethnobotany and Pharmacological Potentials. Emir. J. Food Agric..

[6] Galvis-Pérez Y., Marín-Echeverri C., Franco Escobar C.P., Aristizábal J.C., Fernández M.-L., Barona-Acevedo J. (2020). Comparative Evaluation of the Effects of Consumption of Colombian Agraz (*Vaccinium meridionale* Swartz) on Insulin Resistance, Antioxidant Capacity, and Markers of Oxidation and Inflammation, Between Men and Women with Metabolic Syndrome. BioResearch Open Access.

[7] Quevedo-Rubiano S., Aranda-Camacho Y., Ligarreto-Moreno G.A., Magnitskiy S. (2021). Characterization of the Localized Agri-Food System (SYAL) for the Andean Blueberry (*Vaccinium meridionale* Swartz) in the Boyaca Department, Colombia. Rev. Colomb. Cienc. Hortícolas.

[8] Vargas-Ramella M., Lorenzo J.M., Zamuz S., Valdés M.E., Moreno D., Balcázar M.C.G., Fernández-Arias J.M., Reyes J.F., Franco D. (2021). The Antioxidant Effect of Colombian Berry (*Vaccinium meridionale* Sw.) Extracts to Prevent Lipid Oxidation during Pork Patties Shelf-Life. Antioxidants.

[9] Magnitskiy S.V., Ligarreto G.A. (2011). El Efecto Del Nitrato de Potasio, Del Ácido Giberélico y Del Ácido Indolacético Sobre La Germinación de Semillas de Agraz (*Vaccinium meridionale* Swartz). Rev. Colomb. Cienc. Hortícolas.

[10] Rache Cardenal L.Y., Pacheco Maldonado J.C. (2010). Propagación in vitro de plantas adultas de *Vaccinium meridionale* (Ericaceae). Acta Bot. Bras..

[11] Ehlenfeldt M.K., Polashock J.J., Rowland L.J., Ogden E., Luteyn J.L. (2022). Fertile Intersectional Hybrids of 4x Andean Blueberry (*Vaccinium meridionale*) and 2x Lingonberry (V. Vitis-Idaea). HortScience.

[12] Celis M.E.M., Franco Tobón Y.N., Agudelo C., Arango S.S., Rojano B., Yahia E.M. (2017). Andean Berry (*Vaccinium meridionale* Swartz). Fruit and Vegetable Phytochemicals: Chemestry and Human Health.

[13] SiB Colombia Catálogo de La Biodiversidad de Colombia. https://colecciones.biodiversidad.co/.

[14] Aguilar Garavito M., Ramírez W., Cabrera M. (2014). Restauración Ecológica de los Páramos de Colombia: Transformación y Herramientas para su Transformación.

[15] Madriñán S., Cortés A.J., Richardson J.E. (2013). Páramo Is the World’s Fastest Evolving and Coolest Biodiversity Hotspot. Front. Genet..

[16] Escobar-Trujillo L., Alzate Agudelo G., Echeverry Gómez A., Toro Murillo J., Villegas Londoño C. (2009). Conozcamos y Usemos El Mortiño.

[17] Hernández M.I., Lobo M., Medina C.I., Cartagena J.R., Delgado O.A. (2009). Comportamiento de La Germinación y Categorización de La Latencia En Semillas de Mortiño (*Vaccinium meridionale* Swartz). Agron. Colomb..

[18] Ehlenfeldt M.K., Luteyn J.L. (2021). Fertile Intersectional F1 Hybrids of 4x *Vaccinium meridionale* (Section Pyxothamnus) and Highbush Blueberry, *V. corymbosum* (Section Cyanococcus). HortScience.

[19] Ehlenfeldt M.K., Polashock J.J., Vorsa N., Zalapa J., De La Torre F., Luteyn J.L. (2023). Fertile Intersectional F1 Hybrids of 4x Andean Blueberry (*Vaccinium meridionale*) and 4x American Cranberry (*Vaccinium macrocarpon*). HortScience.

[20] Covarrubias-Pazaran G., Diaz-Garcia L., Schlautman B., Deutsch J., Salazar W., Hernandez-Ochoa M., Grygleski E., Steffan S., Iorizzo M., Polashock J. (2016). Exploiting Genotyping by Sequencing to Characterize the Genomic Structure of the American Cranberry through High-Density Linkage Mapping. BMC Genom..

[21] Campa A., Ferreira J.J. (2018). Genetic Diversity Assessed by Genotyping by Sequencing (GBS) and for Phenological Traits in Blueberry Cultivars. PLoS ONE.

[22] Elshire R.J., Glaubitz J.C., Sun Q., Poland J.A., Kawamoto K., Buckler E.S., Mitchell S.E. (2011). A Robust, Simple Genotyping-by-Sequencing (GBS) Approach for High Diversity Species. PLoS ONE.

[23] Mammadov J., Aggarwal R., Buyyarapu R., Kumpatla S. (2012). SNP Markers and Their Impact on Plant Breeding. Int. J. Plant Genom..

[24] Nyirahabimana F., Shimira F., Zahid G., Solmaz I. (2022). Recent Status of Genotyping by Sequencing (GBS) Technology in Cucumber (*Cucumis sativus* L.): A Review. Mol. Biol. Rep..

[25] Rasheed A., Hao Y., Xia X., Khan A., Xu Y., Varshney R.K., He Z. (2017). Crop Breeding Chips and Genotyping Platforms: Progress, Challenges, and Perspectives. Mol. Plant.

[26] Medina C.I., Lobo Arias M., Patiño M.d.P., Ligarreto G.A., Delgado O.A., Lopera S.A., Toro J.L. (2009). Variabilidad Morfológica en Agraz o Mortiño (Vaccinium meridionale Swartz) en la Zona Altoandina de Colombia.

[27] Medina Cano C.I., Ligarreto Moreno G.A., Vargas Arcila M.O. (2023). Proposal of Descriptors to Study the Variability of *Vaccinium meridionale* Swartz. Rev. Fac. Nac. Agron. Medellín.

[28] Azmat M.A., Khan I.A., Cheema H.M.N., Rajwana I.A., Khan A.S., Khan A.A. (2012). Extraction of DNA Suitable for PCR Applications from Mature Leaves of *Mangifera indica* L.. J. Zhejiang Univ. Sci. B.

[29] Inglis P.W., Pappas M.D.C.R., Resende L.V., Grattapaglia D. (2018). Fast and Inexpensive Protocols for Consistent Extraction of High Quality DNA and RNA from Challenging Plant and Fungal Samples for High-Throughput SNP Genotyping and Sequencing Applications. PLoS ONE.

[30] Bradbury P.J., Zhang Z., Kroon D.E., Casstevens T.M., Ramdoss Y., Buckler E.S. (2007). TASSEL: Software for Association Mapping of Complex Traits in Diverse Samples. Bioinformatics.

[31] Glaubitz J.C., Casstevens T.M., Lu F., Harriman J., Elshire R.J., Sun Q., Buckler E.S. (2014). TASSEL-GBS: A High Capacity Genotyping by Sequencing Analysis Pipeline. PLoS ONE.

[32] Colle M., Leisner C.P., Wai C.M., Ou S., Bird K.A., Wang J., Wisecaver J.H., Yocca A.E., Alger E.I., Tang H. (2019). Haplotype-Phased Genome and Evolution of Phytonutrient Pathways of Tetraploid Blueberry. GigaScience.

[33] Zhidkin R.R., Matveeva T.V. (2022). Phylogeny Problems of the Genus *Vaccinium* L. and Ways to Solve Them. Ecol. Genet..

[34] Powell E.A., Kron K.A. (2002). Hawaiian Blueberries and Their Relatives—A Phylogenetic Analysis of Vaccinium Sections Macropelma, Myrtillus, and Hemimyrtillus (Ericaceae). Syst. Bot..

[35] Kulkarni K.P., Vorsa N., Natarajan P., Elavarthi S., Iorizzo M., Reddy U.K., Melmaiee K. (2020). Admixture Analysis Using Genotyping-by-Sequencing Reveals Genetic Relatedness and Parental Lineage Distribution in Highbush Blueberry Genotypes and Cross Derivatives. Int. J. Mol. Sci..

[36] Manzanero B.R., Kulkarni K.P., Vorsa N., Reddy U.K., Natarajan P., Elavarthi S., Iorizzo M., Melmaiee K. (2023). Genomic and Evolutionary Relationships among Wild and Cultivated Blueberry Species. BMC Plant Biol..

[37] Lawrence M., Huber W., Pagès H., Aboyoun P., Carlson M., Gentleman R., Morgan M.T., Carey V.J. (2013). Software for Computing and Annotating Genomic Ranges. PLoS Comput. Biol..

[38] Lawrence M., Gentleman R., Carey V. (2009). Rtracklayer: An R Package for Interfacing with Genome Browsers. Bioinformatics.

[39] Obenchain V., Lawrence M., Carey V., Gogarten S., Shannon P., Morgan M. (2014). VariantAnnotation : A Bioconductor Package for Exploration and Annotation of Genetic Variants. Bioinformatics.

[40] Yin L., Zhang H., Tang Z., Xu J., Yin D., Zhang Z., Yuan X., Zhu M., Zhao S., Li X. (2020). rMVP: A Memory-Efficient, Visualization-Enhanced, and Parallel-Accelerated Tool for Genome-Wide Association Study. Genom. Proteom. Bioinform..

[41] Frichot E., François O. (2015). LEA: An R Package for Landscape and Ecological Association Studies. Methods Ecol. Evol..

[42] Goudet J., Jombart T., Goudet M.J. (2015). Package ‘Hierfstat’. Estimation and Tests of Hierarchical F-Statistics. https://cloud.r-project.org/.

[43] Yu G., Smith D.K., Zhu H., Guan Y., Lam T.T.-Y. (2017). Ggtree: An R Package for Visualization and Annotation of Phylogenetic Trees with Their Covariates and Other Associated Data. Methods Ecol. Evol..

[44] VanRaden P.M. (2008). Efficient Methods to Compute Genomic Predictions. J. Dairy Sci..

[45] Wang J., Zhang Z. (2022). GAPIT Version 3: An Interactive Analytical Tool for Genomic Association and Prediction. Genom. Proteom. Bioinform..

[46] Kolde R. (2019). Pheatmap: Pretty Heatmaps; CRAN: Vienna, Austria. https://CRAN.R-project.org/package=pheatmap.

[47] Ford-Lloyd B.V., Schmidt M., Armstrong S.J., Barazani O., Engels J., Hadas R., Hammer K., Kell S.P., Kang D., Khoshbakht K. (2011). Crop Wild Relatives—Undervalued, Underutilized and Under Threat?. BioScience.

[48] Jinyuan S., Yu Y., Chong L., Dan L., Du Fang K. (2020). Informing Conservation Strategies with Genetic Diversity in Wild Plant with Extremely Small Populations: A Review on Gymnosperms. Biodivers. Sci..

[49] Ligarreto G.A., del Pilar Patiño M., Magnitskiy S.V. (2011). Phenotypic Plasticity of *Vaccinium meridionale*(Ericaceae) in Wild Populations of Mountain Forests in Colombia. Rev. Biol. Trop..

[50] Zapata I.C., Sepúlveda-Valencia U., Rojano B.A. (2015). Efecto Del Tiempo de Almacenamiento Sobre Las Propiedades Fisicoquímicas, Probióticas y Antioxidantes de Yogurt Saborizado con Mortiño (*Vaccinium meridionale* Sw). Inf. Tecnol..

[51] Quintero-Quiroz J., Galvis-Pérez Y., Galeano-Vásquez S., Marín-Echeverri C., Franco-Escobar C., Ciro-Gómez G., Núñez-Rangel V., Aristizábal-Rivera J.C., Barona-Acevedo J. (2019). Physico-Chemical Characterization and Antioxidant Capacity of the Colombian Berry (*Vaccinium meridionale* Swartz) with a High-Polyphenol Content: Potential Effects in People with Metabolic Syndrome. Food Sci. Technol..

[52] Alam Z., Roncal J., Peña-Castillo L. (2018). Genetic Variation Associated with Healthy Traits and Environmental Conditions in Vaccinium Vitis-Idaea. BMC Genom..

[53] Ferrão L.F.V., Benevenuto J., Oliveira I.D.B., Cellon C., Olmstead J., Kirst M., Resende M.F.R., Munoz P. (2018). Insights Into the Genetic Basis of Blueberry Fruit-Related Traits Using Diploid and Polyploid Models in a GWAS Context. Front. Ecol. Evol..

[54] Ferrão L.F.V., Johnson T.S., Benevenuto J., Edger P.P., Colquhoun T.A., Munoz P.R. (2020). Genome-wide Association of Volatiles Reveals Candidate Loci for Blueberry Flavor. New Phytol..

[55] Nagasaka K., Yamane H., Nishiyama S., Ebihara S., Matsuzaki R., Shoji M., Tao R. (2022). Insights into the Physiological and Molecular Mechanisms Underlying Highbush Blueberry Fruit Growth Affected by the Pollen Source. Hortic. J..

[56] Rodriguez-Bonilla L., Williams K.A., Rodríguez Bonilla F., Matusinec D., Maule A., Coe K., Wiesman E., Diaz-Garcia L., Zalapa J. (2020). The Genetic Diversity of Cranberry Crop Wild Relatives, Vaccinium Macrocarpon Aiton and *V. oxycoccos* L., in the US, with Special Emphasis on National Forests. Plants.

[57] Hokanson K., Hancock J. (1998). Levels of Allozymic Diversity in Diploid and Tetraploid *Vaccinium* sect. Cyanococcus (Blueberries). Can. J. Plant Sci..

[58] Vega-Polo P., Cobo M.M., Argudo A., Gutierrez B., Rowntree J., Torres M.d.L. (2020). Characterizing the Genetic Diversity of the Andean Blueberry (*Vaccinium floribundum* Kunth.) across the Ecuadorian Highlands. PLoS ONE.

[59] Guarnizo C.E., Paz A., Muñoz-Ortiz A., Flechas S.V., Méndez-Narváez J., Crawford A.J. (2015). DNA Barcoding Survey of Anurans across the Eastern Cordillera of Colombia and the Impact of the Andes on Cryptic Diversity. PLoS ONE.

[60] Pennington R.T., Lavin M., Särkinen T., Lewis G.P., Klitgaard B.B., Hughes C.E. (2010). Contrasting Plant Diversification Histories within the Andean Biodiversity Hotspot. Proc. Natl. Acad. Sci. USA.

[61] Sanín M.J., Zapata P., Pintaud J.-C., Galeano G., Bohórquez A., Tohme J., Hansen M.M. (2017). Up and Down the Blind Alley: Population Divergence with Scant Gene Flow in an Endangered Tropical Lineage of Andean Palms (Ceroxylon quindiuense Clade: Ceroxyloideae). J. Hered..

[62] Trénel P., Hansen M.M., Normand S., Borchsenius F. (2008). Landscape Genetics, Historical Isolation and cross-Andean Gene Flow in the Wax Palm, *Ceroxylon echinulatum* (Arecaceae). Mol. Ecol..

[63] Heiser C.B. (1988). Aspects of Unconscious Selection and the Evolution of Domesticated Plants. Euphytica.

[64] Zohary D. (2004). Unconscious Selection and the Evolution of Domesticated Plants. Econ. Bot..

[65] Naflath T., Rajendra Prasad S., Ravikumar R.L. (2022). Population Structure and Genetic Diversity Characterization of Soybean for Seed Longevity. PLoS ONE.

[66] Luteyn J.L. (2002). Diversity, Adaptation, and Endemism in Neotropical Ericaceae: Biogeographical Patterns in the Vaccinieae. Bot. Rev..

[67] Lobos G.A., Retamales J.B., Hancock J.F., Flore J.A., Romero-Bravo S., Del Pozo A. (2013). Productivity and Fruit Quality of *Vaccinium corymbosum* cv. Elliott under Photo-Selective Shading Nets. Sci. Hortic..

[68] Dyukaryeva V., Mallik A.U. (2023). Shade Effect on Phenology, Fruit Yield, and Phenolic Content of Two Wild Blueberry Species in Northwestern Ontario, Canada. Plants.

[69] Ochoa C.I., Sánchez N.Y., Medina C.I., Lobo-Arias M., Galeano P.L., Mosquera A.J., Tamayo A., Lopera Y.E., Rojano B.A., Gaviria C.A. (2009). Propiedades Antioxidantes de Los Frutos de Agraz o Mortiño (*Vaccinium meridionale* Swartz). Perspectivas Del Cultivo de Agraz o Mortiño en la Zona Altoandina de Colombia.

[70] Aguilar C.M., Patino O., González E.A., Nieto M.E., Torres G., Puyana M. (2014). Preliminary Chemical Study and Evaluation of Antioxidant, Antifeedant and Toxic Activity of the Species Pernettya Prostrata (Ericaceae). Rev. Cuba. Plantas Med..

[71] Mezey K. (1943). Pernettya prostrata: Var. pentlandii. Rev. Fac. Med. Vet. Zootec..

