# Supplementary figures and images for: SNP Analysis Reveals Novel Insights into the Genetic Diversity of Colombian Vaccinium meridionale
Source: Genes (Basel). 2025 May 30;16(6):675. doi: 10.3390/genes16060675 (PMC12193593; doi:10.3390/genes16060675)

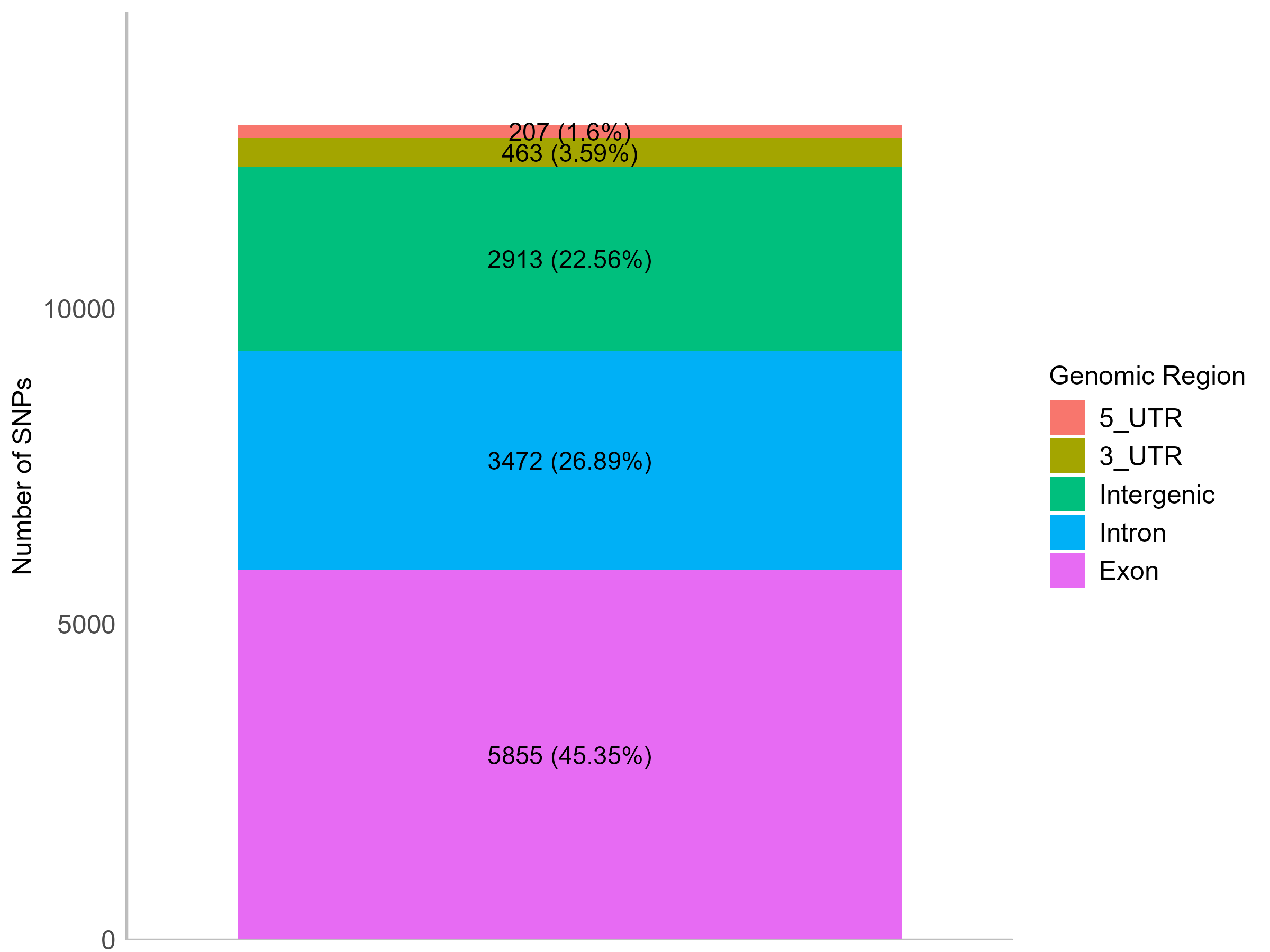

Supplement: Supplementary file 1 [file genes-16-00675-s001.zip › Supplementary Figure S1.tiff]

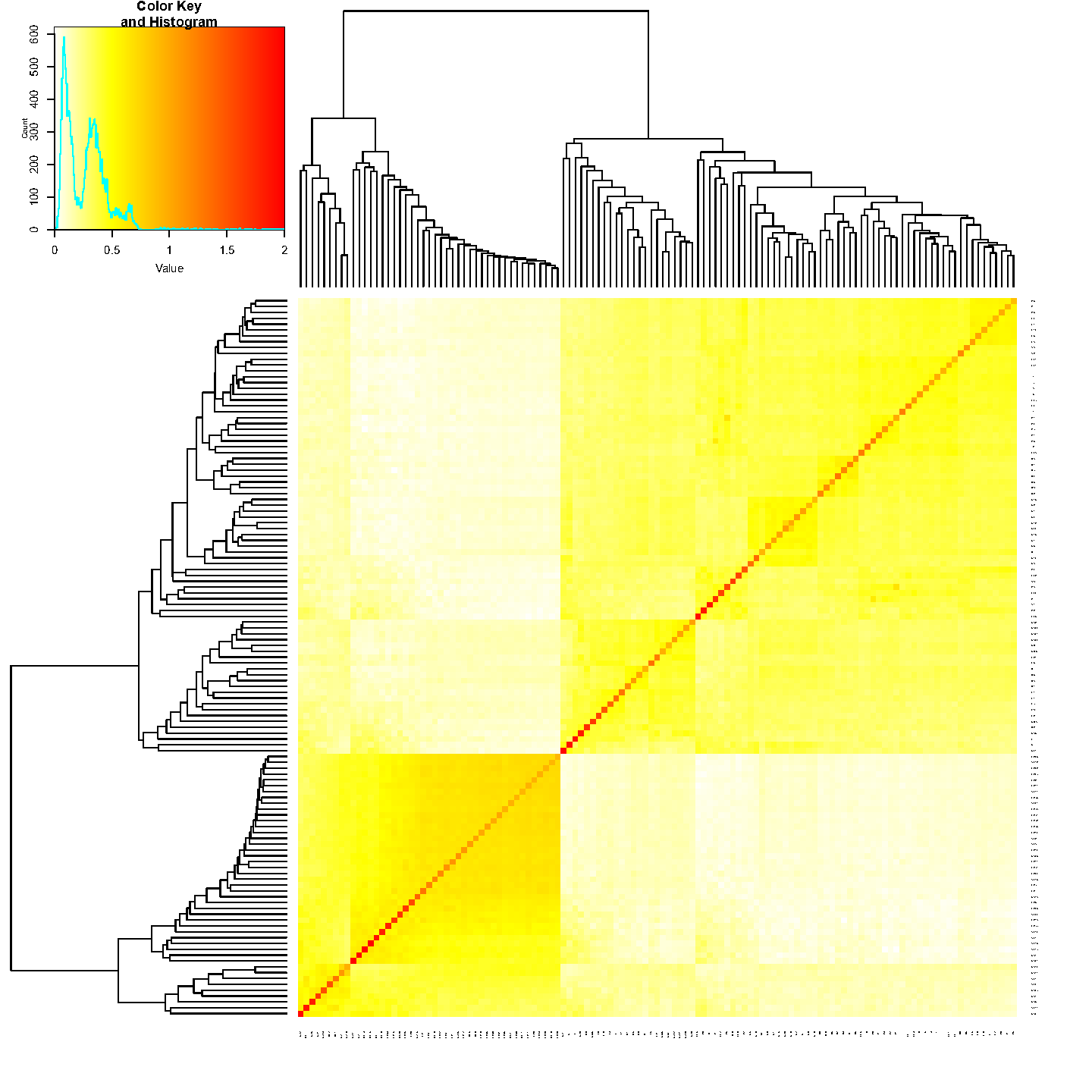

Supplement: Supplementary file 1 [file genes-16-00675-s001.zip › Supplementary Figure S2.tiff]
